# Plant environmental memory: implications, mechanisms and opportunities for plant scientists and beyond

**DOI:** 10.1093/aobpla/plad032

**Published:** 2023-06-06

**Authors:** Gabriela Auge, Valentin Hankofer, Martin Groth, Rea Antoniou-Kourounioti, Irja Ratikainen, Christian Lampei

**Affiliations:** Consejo Nacional de Investigaciones Científicas y Tecnológicas (CONICET), Godoy Cruz 2290, C1425FQB, Ciudad Autónoma de Buenos Aires, Argentina; Facultad de Ciencias Exactas y Naturales, Instituto de Biociencias, Biotecnología y Biología Traslacional (iB3), Universidad de Buenos Aires, Intendente Güiraldez 2160, C1428EGA, Ciudad Autónoma de Buenos Aires, Argentina; Japan Society for the Promotion of Science International Invitational Fellow, Institute for Plant Protection, NARO, 3-1-3 Kannondai, Tsukuba, Ibaraki 305-0856, Japan; Institute of Biochemical Plant Pathology, Helmholtz Munich, Ingolstädter Landstraße 1, 85764 Oberschleißheim, Neuherberg, Germany; Institute of Functional Epigenetics, Helmholtz Munich, Ingolstädter Landstraße 1, 85764 Oberschleißheim, Neuherberg, Germany; School of Molecular Biosciences, University of Glasgow, Sir James Black Building, University Ave, Glasgow G12 8QQ, UK; Department of Biology, Centre for Biodiversity Dynamics, Norwegian University of Science and Technology, Realfagbygget, NO-7491 Trondheim, Norway; Department of Biology (FB17), Plant Ecology and Geobotany Group, University of Marburg, Karl-von-Frisch-Straße 8, 35032 Marburg, Germany

**Keywords:** Ecology, epigenetics, intergenerational effects, memory, metabolism, modelling, plasticity

## Abstract

Plants are extremely plastic organisms. They continuously receive and integrate environmental information and adjust their growth and development to favour fitness and survival. When this integration of information affects subsequent life stages or the development of subsequent generations, it can be considered an environmental memory. Thus, plant memory is a relevant mechanism by which plants respond adaptively to different environments. If the cost of maintaining the response is offset by its benefits, it may influence evolutionary trajectories. As such, plant memory has a sophisticated underlying molecular mechanism with multiple components and layers. Nonetheless, when mathematical modelling is combined with knowledge of ecological, physiological, and developmental effects as well as molecular mechanisms as a tool for understanding plant memory, the combined potential becomes unfathomable for the management of plant communities in natural and agricultural ecosystems. In this review, we summarize recent advances in the understanding of plant memory, discuss the ecological requirements for its evolution, outline the multilayered molecular network and mechanisms required for accurate and fail-proof plant responses to variable environments, point out the direct involvement of the plant metabolism and discuss the tremendous potential of various types of models to further our understanding of the plant’s environmental memory. Throughout, we emphasize the use of plant memory as a tool to unlock the secrets of the natural world.

## Teaser

Plants have an amazing capacity to adapt to their environment and recall prior experiences in order to improve their development and survival. This ‘plant memory’ is a mechanism that allows plants to successfully adjust to changing environments and entails intricate molecular processes with several components and levels. Insights into plant memory and its significance can lead to developments in plant communities in natural and agricultural ecosystems management by integrating modeling with the understanding of ecological, physiological and developmental consequences. This review looks at current breakthroughs in plant memory research, the evolutionary conditions for its development, the extensive molecular networks involved and the significance of plant metabolism. It also emphasizes the possibilities of diverse modeling approaches for unraveling natural world riddles utilizing plant memory as a tool.

## Introduction

Plants have evolved in an ever-fluctuating environment. They are extremely plastic organisms given their need to respond to environmental changes without the ability to move. Plants receive a continuum of information from their environment in their lifetime, and their ability to process this information load is critical to align their growth and development to conditions that favour their survival and proliferation. This requires integration of environmental information and fine-tuning of responses either in a life stage, over their whole life cycle, or beyond—cues perceived in one developmental stage can affect and regulate the timing of subsequent developmental transitions and those of future generations, a sort of *plant environmental memory*. When it helps plants to better balance future developmental trade-offs, plant memory gains adaptive significance. However, plant memory may influence the expression of adaptive traits by constraining future development and survival. As the environmental memory of plants contributes to their realized phenotype, it is shaped by natural selection. Therefore, plant memory commonly varies between populations of the same species at sites that differ environmentally. Consequently, plant memory may play an essential role in local adaptation.

Plants have developed refined molecular responses to match their development and physiological processes with the changes in the environment. As such, in recent years, a rapidly growing body of knowledge has shown that plant memory has an intricate and multilayered molecular basis (Thellier and Lüttge 2013; Crisp *et al.* 2016). This complexity allows plants to store information in the form of molecular modifications (molecules and metabolites) that can be perpetuated and affect the display of phenotypes over time. Either for responses within or across generations, the mechanisms involved need to account for the plastic and reversible nature of plant memory by providing the means to be transient or mitotically/meiotically stable. As environmental memory effects are set and stably transmitted among life stages or generations, their costs should be offset by the benefits of maintaining a lasting molecular function. We can therefore think that dissipation is an important component of plant memory as well. Various abiotic and biotic stressors such as pathogens, extreme temperatures, drought and salinity, encompass the growth regime that changes across different time scales (e.g. diurnally and seasonally) and have the potential to induce memory responses. These environmental cues promote rapid changes in gene expression after exposition with a tissue-specific signal (Zhang *et al.* 2018; Jean-Baptiste *et al.* 2019; C. Zhao *et al.* 2020b). Chromatin structure controls the accessibility of genes for transcription and is regulated by epigenetic modifications, which comprise post-translational modification of histones as well as DNA methylation (Lämke and Bäurle 2017). Epigenetic phenomena are stable and potentially heritable chromatin states that are principally reversible and thus bear the potential for rapid and recurring adaptation to environmental stresses (Lämke and Bäurle 2017; Ashe *et al.* 2021). Thus, epigenetics appear as a strong candidate for the regulation of plant memory. Nonetheless, the main challenges are to identify specific pathways and targets of epigenetic regulation during stress response and adaptation, and to translate such information into new strategies for strengthening and protecting plants against climate change.

Mathematical modelling is a well-established tool that can not only shed light on the relevance and mechanisms underlying plant memory but can also provide a way to predict future plant responses. There are plenty of theoretical models that allow us to infer effects of environmental information integration within generation and a growing number of publications for modelling of transgenerational plasticity (Lawson *et al.* 2015; Peng *et al.* 2020; Crossa *et al.* 2022). The challenge is to put together the available empirical data with those theoretical models to bring forward novel ways in which we can use plant memory as a prediction tool (Yates *et al.* 2018). The potential outcomes and applications are endless—from predicting regional ecological niche shifts for native species facing anthropogenic disturbances, identifying molecular markers that would provide new adaptive traits to crops or native species, to inferring weeds infestation in agricultural ecosystems, just to name a few. The potential for establishing multidisciplinary research efforts and the beneficial outputs from response modelling are extraordinary and undeniable.

In this review, we aimed to explore a body of knowledge that grows day by day to unveil the plant memory secrets. Although fully reviewing the related literature would not fit in a single article, the examples chosen here have the purpose of establishing a background for knowledge on the topic. Firstly, we discuss evidence of the ecological and evolutionary consequences of plants responding to past environments. Then, we try to expose the sophisticated molecular mechanisms—epigenetic, genetic and metabolic—underlying memory effects and shaping within and transgenerational phenotypic plasticity. In both cases, we also highlight the contributions of computational biology to understand the links between environmental cues and responses. Finally, we emphasize the power of mathematical models to help us shed light on the intricacies and predictive potential of plant memory. In this way, we hope to make a strong case for the relevance of plant memory research.

### Regulation of developmental transitions by past environmental cues: ecological consequences

**Figure 1.**
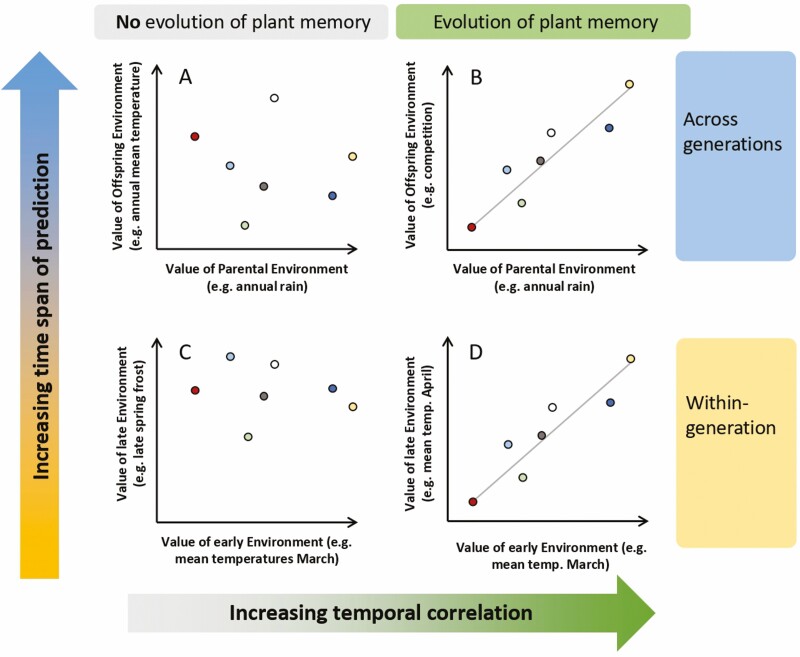
The strength of the temporal correlation between the environmental value at the time of the cue (*x*-axis of subplots) and the time of selection (*y*-axis of subplots) is pivotal for the evolution of an environmental plant memory. Therefore, an adaptive plant memory can evolve only when the temporal correlation is sufficiently strong (B, D vs. A, C). The time span between the environmental cue and the time of selection relative to the life span of the plant determines whether the memory is conserved across a plant’s life or across generations (A, B vs. C, D). In general terms, when the time span of prediction is short, but the environmental correlation is strong, within-generation memory can evolve (D vs. C); while when that span is long, transgenerational memory is favoured (B vs. A). The subplots show potential values for example of environments. (A) Annual rain in one year is usually not correlated with the mean annual temperature in a later year and accordingly is a poor predictor for offspring heat response. However, (B) in rain-limited habitats, rain is a good predictor of next year’s seedling density (i.e. temporal correlation between two different environmental variables) and accordingly modulates offspring germination via a trans-generational environmental memory. (C) The increasing spring temperatures due to climate change frequently cause early budbreak but represent a poor predictor for late spring frost, resulting in severe frost damage due to missing within-generation plasticity to prepare for the frost. However, (D) generally, increasing temperatures after winter represent a reliable cue for the start of the growing season (i.e. temporal autocorrelation within the time series of temperature values), so that plants can find the best time for budbreak using within-generation memory. The points represent hypothetical data points from different years or geographic coordinates with point colour as year/site identifier.

An environmental memory evolves under a particular environmental condition (Figure 1). Most importantly, the environmental cue must predict the selection scenario met at a later developmental stage or in a later generation and thus provide an opportunity to optimize the phenotype before selection (Ezard *et al.* 2014; Leimar and McNamara 2015; Auge *et al.* 2017). This means that a correlation across time must exist between the environmental cue and the environmental value at the time of selection (Figure 1). Theoretically, this selection scenario is met when environmental variables known as selective agents, such as temperature or precipitation, are temporally autocorrelated. The term ‘temporal autocorrelation’ describes the phenomenon when a time series shows similar values (e.g. temperature) at regular intervals. In other words, environmental memory is generally favoured when parents and offspring inhabit similar environments and when there is any form of restriction or cost preventing the offspring from immediately sensing the selective environment they will experience (Silva *et al.* 2021). Lack of similarity in selective environments can be due to low autocorrelation in environmental factors or high dispersal away from the local microclimate. Although there is no evolutionary advantage for within-generation plasticity over transgenerational plasticity if all costs, benefits and error rates are equal, within-generation plasticity evolves faster and can therefore be expected to appear more often (Dury and Wade 2020). However, a recent study has shown that some traits more than others are conducive to the evolution of parental effects, with viability selection being more likely to produce evolution of such effects (Kuijper and Johnstone 2021). In accordance with this and considering climatic conditions in the US territories, Colicchio and Herman (2020) demonstrated that autocorrelation of selective environments can be found on earth frequently. Specifically, they showed that temporal autocorrelations in long-term rain and temperature data supported the evolution of transgenerational memory in an organism with a one-year life cycle according to optimality models (Colicchio and Herman 2020). These results suggest that the environmental preconditions favouring the evolution of an environmental memory do not present a special case but are commonly met in nature.

One well-known example of temporal autocorrelation in nature is a directional change in climate, such as the current atmosphere warming, known as global climate change. In this case, the temperature experienced by the parental plant can be a better predictor of the temperature experienced by offspring than the long-term average temperature over many years to which the local population is adapted historically. For example, a conceptual model suggested that an environmental memory advancing flowering after parental heat exposure in *Arabidopsis thaliana* evolved when selection pressure for early flowering was high and autocorrelated, equivalent to a steadily advancing onset of summer heat that terminates the growing period, such as predicted by the projections of climate change (Groot *et al.* 2017). Related to this environmental memory is the earlier leaf flush after parental experience of elevated temperatures, as observed in poplar (*Populus deltoides* and *P. trichocarpa*, Dewan *et al.* 2018) and oak (*Quercus robur*, Dewan *et al.* 2020). However, for *A. thaliana*, in temperate environments, where summers provide sufficient precipitation, early flowering does not provide the same benefit, and accordingly, the environmental memory has not evolved (Groot *et al.* 2017). Only a few studies from other plants are available for comparison. In *Plantago lanceolata* (Lacey 1996) and in rice (*Oryza sativa* L., Koumoto *et al.* 2018), flowering was delayed after parental exposure to warm temperatures. However, in both studies, it remained unresolved whether this environmental memory represents a benefit for the progeny in the plant’s natural environment.

Apart from long-term climatic oscillations, such as climate change (Colicchio and Herman 2020), the year-to-year weather variation is largely stochastic. However, stochastic variation of the abiotic environment can predictably affect the biotic environment, which in turn can contribute strongly to the evolution of an environmental memory (Lampei *et al.* 2017). In the annual Mediterranean plant, *Biscutella didyma*, mothers experiencing higher water availability produced more dormant seeds (Lampei *et al.* 2017). This is an environmental memory that likely evolved due to density dependence. The mothers ‘keep’ part of their seeds in the seed bank for a later chance, because high numbers of seeds are produced in a rainy year, causing high seedling density, i.e. competition, in the next year. An important aspect here is that this environmental memory did not evolve due to an autocorrelation in a single variable. The parental and the offspring environments (seasons) received very different amounts of rain. However, the rain experienced by the mother is temporally correlated with a different variable that also determines offspring success, namely competition intensity. Notably, the strength of the temporal correlation between the randomly varying amounts of yearly rain and the seedling density in the following year accurately predicted the strength of the environmental memory for each population, suggesting that this correlation was closely connected with the evolution of the environmental memory (Lampei *et al.* 2017). This suggests that temporal autocorrelation maps of rain and temperature data, such as those presented by Colicchio and Hermann (2020) even underestimate the potential for the evolution of environmental memories in natural environments, because they cannot incorporate the potential of biotic selective agents.

Furthermore, plants can prepare their offspring for a competitive environment also beyond germination. In *Taraxacum brevicorniculatum* offspring of parents exposed to stronger competition were better prepared for competition through various trait changes, like faster development and increased investment in roots (Puy *et al.* 2021). Further, the offspring grew taller and produced lighter seeds, which may improve dispersal. However, despite improved dispersal, seeds are likely to drop not far from the mother, and the competition environment is likely unchanged in the following year. In other words, the environmental conditions are spatially autocorrelated, and through limited dispersal, this spatial autocorrelation turns into a temporal environmental autocorrelation between generations.

Also, herbivores are not randomly distributed in space, and when the mother plant is exposed to herbivory, this will most likely be valid also for the offspring. In *Nicotiana attenuate*, the combined experience of above-ground and below-ground herbivory in the parental generation increased offspring herbivory resistance, and parental below-ground herbivory increased root biomass in offspring (Kafle and Wurst 2019). How plants can improve their defence was observed in wild radish (*Raphanus sativus*), which, after parental herbivore exposure, showed an improved physical defence via a higher trichome density and an increased chemical defence through producing more glucosinolates in the offspring that had not encountered herbivory themselves (Sobral *et al.* 2021). Similarly, the annual plant *Mimulus guttatus* reduced offspring herbivory in the field following experimental parental wounding through increased trichome density (Colicchio 2017). Together, these examples suggest that an environmental memory preparing offspring for herbivory encountered by the parents may be a widespread phenomenon across several plant families. However, Colicchio (2017) incorporated several accessions of *M. guttatus*, some of which produced fewer trichomes after parental wounding, suggesting that the mode of repeated occurrence of herbivores may not be uniform across the landscape. Therefore, it is generally advisable to incorporate more than one accession or population when investigating the environmental memory of a species, as genotype × environment interactions are commonly observed (Colicchio 2017; Groot *et al.* 2017; Lampei *et al.* 2017; Alvarez *et al.* 2020; Deng *et al.* 2021).

In addition to the existence of correlations across time, another important aspect is the length of the period between the environmental cue and the predicted selection event (Auge *et al.* 2017) or the time it takes for the plant to adjust its phenotype (Burton *et al.* 2022). Naturally, predictions are usually more accurate when bridging a short rather than a long period. The evolution of an environmental memory between generations can be more likely to evolve in short-lived rather than in long-lived species, and the evolution of environmental memory should be understood as part of a co-evolutionary process with life-history traits (Ratikainen and Kokko 2019). In accordance, Yin *et al.* (2019) showed in a meta-analysis that environmental transgenerational memory was more frequently found in annual than in perennial plants. The authors reasoned that long-lived species mostly experience temporal correlations spanning a much shorter period than their lifespan. They concluded that the evolution of such environmental memory requires a good match between the lifespan and the timescale of the prediction. However, for long-lived species, such as trees, the environmental information gathered by the parents during the last years before seed production is most likely a better environmental cue for their offspring than the selection regime when the parents were saplings. In *Pinus sylvestris*, the survival of offspring from a long-term irrigation project was higher the longer the parents had been exposed to drought (Bose *et al.* 2020). Accordingly, there is more and more evidence from several tree species for the important contribution of an environmental memory for seedlings and saplings (Dewan *et al.* 2018, 2020; Bose *et al.* 2020). These results demonstrate that environmental memory can also benefit long-lived species.

Together, the above-listed examples reveal that plants can evolve environmental memories in response to abiotic or biotic environmental cues (Figure 1). Further, even when the environmental cue is abiotic, the predicted selection event can be biotic, such as density dependence. In their natural environment, plants are exposed to a complex selection scenario involving multiple players (Mertens *et al.* 2021). However, tests of environmental memory and its consequences are rarely conducted under natural conditions (but see Colicchio, 2017). Therefore, these controlled experiments possibly overlook important interactions of simultaneously received environmental cues. For example, in *A.**thaliana*, stress treatments, like simulated herbivory or shading, triggered an adaptive environmental memory in the offspring. However, this was not the case when the treatments were combined (Lampei 2019). Instead, the plants responded directly to the treatment combinations through within-generation plasticity. Notably, in this study, the environmental memory adapted the offspring phenotype to the expected environment with nearly no fitness costs. Contrarily, the direct plastic response to the combined treatments significantly delayed flowering (Lampei *et al.* 2019). This suggests that plants may choose different strategies associated with differing costs, depending on the predictability of the environmental cue. However, more studies are needed to test the ecological and evolutionary consequences of environmental memories under more complex and realistic conditions.

### Molecular mechanisms involved in the regulation of plant memory

The wealth of evidence on plant memory suggests a complex regulation at the molecular level. The fine regulation displayed by environmental memory effects is likely mediated by a combination of genetic and epigenetic elements, which result in a range of within and transgenerational plastic responses. These underlying molecular mechanisms are often intricate, and we have only started to unravel them for intergenerational environmental memory (see also Sharma *et al.* 2022). However, some within-generation memories are well studied and give useful insights into the complex molecular regulation of the plants’ interaction with their environment.

Probably the most studied within-generation memory mechanism in plants is the one involving the regulation of flowering by overwintering, a process called vernalization. The *A. thaliana* MADS box transcription factor *FLOWERING LOCUS C* (*AtFLC*) is a central inhibitor of flowering and serves as a key developmental checkpoint where multiple signalling pathways converge (Shindo *et al.* 2005; Deng *et al.* 2011; Méndez-Vigo *et al.* 2015; Whittaker and Dean 2017; Buzas *et al.* 2021). *AtFLC* regulation is a multilayered process, ranging from transcriptional regulation to epigenetic silencing (Choi *et al.* 2011; Yang *et al.* 2017; Antoniou-Kourounioti *et al.* 2018; Qüesta *et al.* 2020), allowing the fine-tuning of *AtFLC* expression by environmental cues. Most importantly, *AtFLC* silencing by epigenetic mechanisms after the exposure to cold is stable in *A. thaliana* plants, even after temperatures rise, prolonging the memory of winter until repression is reset during fertilization (Shindo *et al.* 2005; Crevillén *et al.* 2014). This repression is controlled by the Polycomb Repressive Complex 2 (PRC2), which modifies histone H3 lysine 27 residues by trimethylation (H3K27me3) and promotes a perpetuated silencing state of the *AtFLC* locus (Angel *et al.* 2011). Mathematical modelling has furthered our understanding of this mechanism, showing it involves a digital memory switch, in which gradual repression of a gene at the population level is achieved by an ON-to-OFF transition of that gene in each cell happening asynchronously (Angel *et al.* 2011). Later work into the H3K27me3 states involved in the memory of winter revealed the need for three separate such states (depending on the location of the H3K27me3 mark along the gene and the presence of additional proteins), each respectively responsible for the establishment, consolidation and perpetuation of the epigenetic memory (Yang *et al.* 2017; Qüesta *et al.* 2020; Lövkvist *et al.* 2021). Combining lab and field experiments, along with modelling, has shed light on the differences in environmental memory regulation between annual (*A. thaliana*) and perennial species (*Arabidopsis halleri*), which display irreversible and reversible vernalization effects, respectively (Antoniou-Kourounioti *et al.* 2018; Nishio *et al.* 2020). The latter revealed the complex interconnectivity of histone methylation marks (H3K27me3 and H3K4me3) at different locations on the *AhgFLC* gene. This shed light into a complex system at *AhgFLC* with a directionality in the response to seasons (a ‘ratchet-like character’) so that the gene was not sensitive to winter cold until it had been fully reset by summer. This property is important for a perennial species such as *A. halleri*, making it able to perform the full seasonal cycle. *AtFLC* is also regulated by at least four different long non-coding RNAs (lncRNA)—*COOLAIR*, *COLDAIR*, *COLDWRAP* and *ASL*—with different modes of action but sharing their silencing function (Heo and Sung 2011; Csorba *et al.* 2014; Shin and Chekanova 2014). For example, the antisense transcripts collectively called *COOLAIR* silence *AtFLC* by physically associating with the *AtFLC* locus and affecting the H3K36 trimethylation, in consequence modifying the chromatin state around the locus (Csorba *et al.* 2014). The interaction between all these factors (histone and chromatin modifications and transcriptional regulation) in a sort of sequential, almost redundant regulatory mechanism, is necessary to keep the memory of winter within *A. thaliana*’s life cycle. *AtFLC* is reactivated (i.e. the vernalization silencing effect is dissipated) during embryogenesis and its expression is induced during seed maturation through the recruitment of chromatin modifiers mediated by the transcription factor ABSCISIC ACID-INSENSITIVE3 (ABI3), which binds to a *cis*-acting cold element switching the *AtFLC* locus into an active state (Xu *et al.* 2022). Similar mechanisms of stable regulation of flowering repressors are found in cereals, for which vernalization effects were first described (Sharma *et al.* 2020). The multilayered regulatory mechanism described here would provide an almost fail-safe regulation of flowering time. But, what happens if winter is not strong or long enough to establish the conditions for environmental memory? More layers of regulators are added, such as that of flowering regulation mediated by the alternative splicing of the gene *AtFLM.* Post-transcriptional processing of *AtFLM* renders two splice variants with proposed opposing functions (Lutz *et al.* 2017). This fallback mechanism ensures the repressive protein splice variant *FLM*-β, expressed in cooler environments, is counteracted by the competitive *FLM*-δ, in this way releasing the repression on flowering under ambient temperatures and allowing flowering to occur independently of *AtFLC* (Posé *et al.* 2013). Even though it is known that *AtFLM* does not interact genetically or phenotypically with *AtFLC*, which excludes *AtFLM* from playing a role in the memory of winter in *A. thaliana* (Scortecci *et al.* 2003), its effect on flowering time regulation demonstrates plants have evolved additional mechanisms to overcome the lack of environmental memory establishment and maintenance.

While vernalization depends on exposure to cold temperatures, heat induces another type of molecular thermomemory that primes plants against recurring heat stress (Balazadeh 2022). Heat stress leads to transcriptional activation of heat-inducible genes that remain activated for a few days after recovery from the initial stress (type I transcriptional memory) or become re-activated more efficiently upon a recurring stress (type II memory) (Oberkofler *et al.* 2021; Balazadeh 2022; Perrella *et al.* 2022). To maintain transcriptional memory and therefore ensure thermotolerance, a heteromeric complex containing HEAT SHOCK TRANSCRIPTION FACTOR A2 and A3 (HSFA2 and HSFA3, respectively) is specifically required for memory-gene expression after heat exposure (Friedrich *et al.* 2021). HSFA2 mediates H3K4 trimethylation (H3K4me3) at thermomemory genes after heat stress in a ‘hit and run fashion’, i.e. H3K4me3 enrichment is sustained beyond transient HSFA2 enrichment at chromatin (Lämke *et al.* 2016). Employing time-course ChIP, Friedrich *et al.* (2021) subsequently showed that HSFA2 and HSFA3 directly bind to memory gene promoters such as *HSP22* and *APX2*, with enrichment of HSFA3 still detected after 28 h into the recovery phase. Conclusively, H3K4me3 is a hallmark of thermomemory as it enables sustained activation of type I memory genes (HSFA2/3-dependent) as well as enhanced re-activation of type II memory genes (HSFA2-dependent) (Lämke *et al.* 2016; Friedrich *et al.* 2021; Balazadeh 2022).

In addition to short-term thermotolerance, severe heat stress can manifest in early flowering and reduced pathogen defence through inhibition of post-transcriptional gene silencing (PTGS) in stressed plants, as well as in non-stressed offspring (Zhong *et al.* 2013; Li *et al.* 2014). Recently, the transgenerational phenotype has been linked to a positive feedback mechanism between heat-induced HSFA2 and transcriptional activation mediated by the H3K27me3 demethylase RELATIVE OF EARLY FLOWERING 6 (REF6) and the chromatin remodeller BRAHMA (BRM) (Liu *et al.* 2019). Accordingly, HSFA2 binds to heat shock elements (HSEs) in the promotor regions of *REF6* and *BRM*, and those in turn target HSFA2, leading to H3K27me3 reduction and HSFA2 up-regulation, which is inherited by the non-stressed progeny. Moreover, HSFA2 activates an E3 ubiquitin ligase SUPPRESSOR OF GENE SILENCING 3 (SGS3)-INTERACTING PROTEIN 1 (SGIP1), which leads to degradation of SGS3 and, consequently, to the inhibition of PTGS. Ultimately, reduced SGS3-dependent production of transacting small interfering RNAs (tasiRNAs) contributes to the upregulation of HEAT-INDUCED TAS1 TARGET 5 (HTT5), which drives transgenerational thermomemory phenotypes. In summary, the heritable REF6/BRM-mediated H3K27me3 demethylation and HSFA2 activation may cooperate to maintain a transgenerational ‘ON state’ of thermomemory genes (Liu *et al.* 2019).

A well-studied short-term, within-generation, memory process in plants is the priming of the immune system by biotic stresses. Plenty of examples show that after a first challenge by a stressor, plants elicit systemic immune responses and can memorize previous attacks to protect themselves, and sometimes those plants nearby, from future challenges by pathogens and herbivores (Conrath *et al.* 2015). This priming of the plant immune response is mediated by various mechanisms of differing nature, ranging from transcriptional hormone signalling activation to epigenetic and post-translational mechanisms, and the interaction between some of them. The involvement of DNA methylation was proposed by different studies showing enhanced susceptibility or resistance of DNA methylation mutants to necrotrophic or biotrophic pathogens, respectively (López *et al.* 2011; Dowen *et al.* 2012; Luna *et al.* 2012), but the underlying mechanisms are still poorly understood. An advancement came from a recent study by Halter *et al.* (2021) that looked into the *cis*-regulatory effects of DNA methylation at transposable elements (TEs) on nearby pathogen defence genes. In this example, active demethylation of TEs inside the regulatory region of *TNL RESISTANCE METHYLATED GENE1* (*RMG1*) by 5-methylcytosine DNA demethylase REPRESSOR OF SILENCING 1 (ROS1) was required for basal resistance against the pathogenic bacterium *Pseudomonas syringae* pv. tomato strain DC3000 (Pst) (Deleris *et al.* 2006; Halter *et al.* 2021). Indeed, the *RMG1* promoter holds two remnant RC/Helitron TEs, of which the proximal repeat *ATREP11* (*A. thaliana REPEAT 11*) is hypermethylated in *ros1* mutant plants (Yu *et al.* 2013; Halter *et al.* 2021). This is in accordance with ROS1 protecting genes from silencing by limiting DNA methylation spread at TE boundaries via 21–24 nt small interfering RNAs (siRNAs) as part of the RNA-directed DNA methylation pathway (RdDM) (Tang *et al.* 2016; Halter *et al.* 2021). Accordingly, *DICER-LIKE 2* (*dcl2*) and *dcl3* loss of function mutants, which are impaired in the production of 23 and 24 nt siRNAs, restored *RMG1* expression in the *ros1* genetic background (Halter *et al.* 2021). This study highlights that the balance of active DNA methylation and demethylation is vital for pathogen defence in *Arabidopsis* and that perturbations at regions of intermediate stability may lead to a disadvantageous physiological stress responses that can affect plant immunity memory as well.

Simultaneously, stress-induced TE mobilization has the potential to yield new adaptive traits by introducing genetic variation, including the insertion of response elements into gene regulatory regions (Quadrana *et al.* 2016, 2019; Roquis *et al.* 2021). COPIA78/ONSEN is an example of a stress-activated transposon in *A. thaliana* that contains *cis*-regulatory sequences and exhibits similar stress-responsive expression patterns as proximal protein-coding genes (Ito *et al.* 2011; Roquis *et al.* 2021; Nozawa *et al.* 2022). While a growing number of studies discover *cis*-regulatory sequences in plant genomes, there are still open challenges on their characterization, functional evaluation (promoters, transcriptional enhancers, silencers and insulator elements) and the identification of their target genes (Schmitz *et al.* 2022). Further insight is also needed on the causality of DNA methylation on TE activation in response to stress stimuli and its role in stress memory.

Long-term, transgenerational memory also highlights the complex interaction between genetic and epigenetic regulation (Alvarez *et al.* 2020, 2021). A growing number of reports show that especially DNA methylation is a good candidate as response mediator to environments from past generations. Exemplary for the interaction of genetic and epigenetic variation in the context of environmental adaptation are different genome-wide association studies using wild *A. thaliana* accessions. One of these studies looked for alleles associated with climate variables from the collection sites and found that natural variation at *CHROMOMETHYLASE2* (*CMT2*) is associated with temperature seasonality (Shen *et al.* 2014). In line with the function of CMT2, accessions carrying the allele with a nonsense mutation associated with higher temperature seasonality more often showed reduced methylation in the CHH context than accessions with the wild-type *CMT2* allele. Moreover, artificial *cmt2* mutants tolerated heat better than wild-type plants (Shen *et al.* 2014). These results agreed with another study that identified a strong association between natural variation at *CMT2* and CHH methylation patterns, as well as correlations between CHH methylation and growth temperature in Swedish *Arabidopsis* accessions, which collectively support the idea that natural selection of genetic determinants of DNA methylation patterns contributes to climate adaptation (Dubin *et al.* 2015; Kawakatsu *et al.* 2016). Compared to mammals, plant gametes form post-embryonically, which increases the possibility of heritable adaptive DNA methylation changes in response to the growth environment (Grossniklaus 2011; Schmid *et al.* 2018). However, it remains controversial if DNA methylation itself is stable enough to be subject to selection and thus have an impact on adaptation at the population level (Schmid *et al.* 2018). Consistent with a confirmation of its contribution and the examples listed above, large-scale patterns of DNA methylation were constant throughout the year for *A. halleri* in its natural habitat (Ito *et al.* 2019), and another study demonstrated that environmental stress may lead to long-lasting changes in DNA methylation (Zhang *et al.* 2016).

In the context of DNA methylation, siRNA can also serve as long-term messengers in plants. Seed size, a proxy for maternal investment in progeny production that is highly sensitive to maternal environmental conditions, is regulated by the RdDM pathway (Kirkbride *et al.* 2019). Mutants in this pathway show spatial and temporal misregulation of *AGAMOUS-LIKE* genes such as *AGL40* and *AGL91*, which results in increased endosperm cell expansion and seed size in *A. thaliana* (Kirkbride *et al.* 2019). Seed size constrains seed responses to environmental changes and is correlated with altered vigour and quality in agriculturally relevant species, as well as establishment potential in natural environments (Zhang *et al.* 2016; Catford *et al.* 2019; Wijewardana *et al.* 2019; Fernández‐Pascual *et al.* 2020). The RdDM pathway also mediates the response of progeny seeds to the environment experienced by maternal plants during their whole life cycle (Iwasaki *et al.* 2019; Authier *et al.* 2021) indicating DNA methylation is a key player in the regulation of responses to environments of previous generations.

In accordance with the previous examples, recurring hyperosmotic stress over multiple generations results in higher survival rates that correlated with changes in DNA methylation patterns in *Arabidopsis* (Wibowo *et al.* 2016). Importantly, one hyperosmotic stress-free generation resulted in the loss of transgenerational salt tolerance in the progeny, which was accompanied by the resetting of DNA methylation patterns (Wibowo *et al.* 2016). Enhanced tolerance to hyperosmotic stress was primarily inherited through the female germline as male gametes passed through epigenetic reprogramming mainly mediated by DEMETER (DME) DNA glycosylase activity (Ibarra *et al.* 2012; He *et al.* 2019). Moreover, multiple differentially methylated regions (DMRs) were linked to hyperosmotic stress response genes showing that their activation upon stress depends on their methylation status (Wibowo *et al.* 2016).

In addition to somatic defence priming and reminiscent of the intergenerational salt stress memory described above (Wibowo *et al.* 2016), previous studies highlighted the potential of transgenerational acquired resistance (TAR), or transgenerational induced resistance, passed from diseased plants to progeny (Luna *et al.* 2012; Stassen *et al.* 2018; López Sánchez *et al.* 2021). Employing TAR, plants can transmit acquired resistance, a type of systemic adaptive immunity, to following generations, leaving progeny better equipped against pathogens with a similar infection strategy (Luna *et al.* 2012; Slaughter *et al.* 2012; Stassen *et al.* 2018). Since TAR has been linked to changes in DNA methylation and histone modifications, which are often reversible (Zhu *et al.* 2016; López Sánchez *et al.* 2021), epigenetic mechanisms have the potential to transmit along pathogen resistance transiently and to terminate in stress-free generations to avoid associated costs, therefore making TAR an ecologically plausible mechanism (Stassen *et al.* 2018).

**Figure 2.**
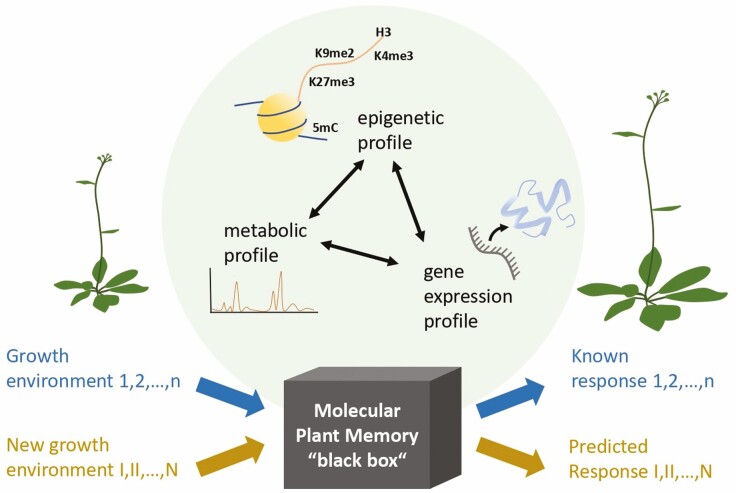
Plant memory is regulated by the interaction of different genetic, epigenetic and metabolic mechanisms (‘shaded sphere’). The role of each of these components in the establishment, transmission, maintenance and dissipation of within- and transgenerational effects is dependent on the environmental stimuli and the kind of memory involved. Yet, it is not always fully understood how plant memories that correlate with enhanced adaptation to recurring environmental stimuli are retained (‘dark box’). To help bridging this knowledge gap, we can use the empirical data on the contributing molecular components of a known response (‘upper arrows’) to inform and help train mathematical models that make predictions about future physiological, ecological and evolutionary responses of plants (‘bottom arrows’) living in natural and agricultural ecosystems. Thus, the power of plant memory knowledge can be unleashed and can be used to guide agriculture, conservation and management efforts.

Altogether, these examples point to a finely regulated and intricate network of layered mechanisms that are involved in the establishment, transmission, response and dissipation of environmental memory in plants with full adaptive potential (Figure 2).

### The metabolism as an underestimated mechanism regulating plant memory

To add more layers of complexity to the regulation of plant memory, another likely element in the regulatory mechanism of environmental memory is the metabolism. Here, little is known so far about the involvement of metabolism in long-term environmental memory, so we present examples associated with within-generation environmental memory to outline the principles. Plant metabolic pathways are complex and highly adaptive to environmental change (Lindermayr *et al.* 2020). Activation of defence genes against pathogens as part of transcriptional reprogramming in systemic acquired resistance involves well-described hormone-signalling networks and secondary metabolites to prevent pathogen propagation (Bolton 2009; Vlot *et al.* 2021; Escaray *et al.* 2022). Consequently, pathogen response leads to an increased demand for metabolic intermediates (Bolton 2009; Escaray *et al.* 2022). Recent studies uncovered a link between methionine (Met) metabolism and plant defence by characterizing pigmR-interacting and chitin-induced protein 1 (PICI1) (Escaray *et al.* 2022; Zhai *et al.* 2022). PICI1 promotes disease resistance against rice blast fungus *Magnaporthe oryzae* in rice by stabilizing the enzyme methionine synthase (METS1) through deubiquitination—a protein providing Met, the precursor of ethylene which functions in plant immunity (Escaray *et al.* 2022; Zhai *et al.* 2022). Furthermore, overexpression of *METS1* in *A. thaliana* promoted disease susceptibility to *P. syringae* DC3000, as well as overall higher DNA methylation levels (González and Vera 2019). Together, these results indicate a connection between pathogen defence, DNA methylation and Met metabolism, with potential consequences for long-lasting or primed responses to future pathogen challenges.

Similarly, proper DNA methylation requires additional enzymes involved in Met metabolism, including S-ADENOSYLMETHIONINE SYNTHETASE, S-ADENOSYLHOMOCYSTEINE HYDROLASE and METHYLENETETRAHYDROFOLATE DEHYDROGENASE/METHENYLTETRAHYDROFOLATE CYCLO-HYDROLASE 1 (MTHFD1, Li *et al.* 2011; Rocha *et al.* 2015; Groth *et al.* 2016). MTHFD1 is an enzyme of the cytosolic folate-mediated one-carbon metabolism, which provides 5-CH3-THF as a substrate for METS1-catalysed re-methylation of homocysteine (Hcy) to Met. This reaction combines two essential premises for cellular methylation reactions: first, Met synthesis, which is further processed to S-adenosylmethionine (SAM), the methyl group donor for transmethylation reactions to cytosine in the DNA; and second, removal of Hcy, the product of S-adenosylhomocysteine (SAH) hydrolysis. SAH is the byproduct of transmethylation reactions and is known to competitively inhibit DNA (and other) methyltransferases (de la Haba and Cantoni 1959; Borchardt *et al.* 1974). The *MTHFD1* missense mutant *mthfd1-1* impairs one-carbon metabolism, resulting in SAH and Hcy accumulation as well as genome-wide loss of DNA methylation, reduced histone H3K9 dimethylation (H3K9me2) and de-repression of TEs (Groth *et al.* 2016). The importance of enzymes such as MTHFD1 and METS1 in providing folate-mediated one-carbon for methylation reactions illustrates the need for profound analyses of regulatory mechanisms and dynamics at the interface of metabolism and epigenetic regulation in plants. One intriguing consequence of the dependence of epigenetic regulation on metabolic intermediates is that genes involved in the turnover of these metabolites (e.g. SAM) themselves may be under epigenetic regulation in response to environmental factors, thereby creating a self-regulatory feedback loop. Functional examples supporting this idea are still lacking, but intriguingly genes involved in sulfur uptake, including sulfate transporter genes *SULTR1;1* and *SULTR1;2*, showed DNA hypomethylation in regulatory regions and constitutive activation in mutant plants that were impaired in SAM homeostasis due to mutation of *SERINE HYDROXYMETHYLTRANSFERASE 7*, suggesting that sulfur metabolism is linked to DNA methylation through feedback-regulation mediated by SAM levels (Huang *et al.* 2016).

In addition, by affecting the redox state of cells, reactive oxygen species (ROS) regulate the response of genes coding histone modification and chromatin remodelling proteins (Dietzel *et al.* 2015). Proline metabolism regulates root development by controlling ROS accumulation (Bauduin *et al.* 2022), a likely mechanism for the regulation of flowering time by proline as well (Mattioli *et al.* 2022). Proline regulates flowering time regulation by affecting *AtFLC* expression: proline-deficient mutants show a significant upregulation of *AtFLC* and downregulation of downstream integrators and meristem identity genes (Mattioli *et al.* 2022). When those mutant plants are vernalized, *AtFLC* expression is repressed and flowering is promoted. Although it is also likely that the proline-deficient mutant phenotype leads to a pleiotropic effect resulting in overall late development, which could be overcome by accelerating flowering time by vernalization, whether this metabolic mechanism can contribute to the establishment of a within-generation memory state by epigenetically silencing *AtFLC* remains to be understood.

There is some evidence pointing to the metabolism as mediator of transgenerational memory. Water stress (drought and waterlogging) induces oxidative stress by modulating the accumulation of ROS (Deng *et al.* 2012; Boaretto *et al.* 2014). In *P. lanceolata*, offspring experiencing drought and waterlogging showed increased performance (photosynthetic pigment accumulation and upregulation of genes coding antioxidative machinery) when mother plants experienced those same stresses themselves (Lukić *et al.* 2023). This adaptive response could decrease water stress-associated oxidative damage in the offspring to protect the photosynthesis function and overall tolerance to stress.

This evidence highlights the involvement of metabolism in the regulation of some of the plant memory-associated mechanisms (Figure 2). Responses to biotic and abiotic stresses can be mediated by metabolic imprints (metabolic states perpetuating long after stress recovery) and priming (delayed adjustment of metabolite levels to pre-stress state), providing a long-lasting and systemic pathway of regulation (Schwachtje *et al.* 2019). Given the high sensitivity of the metabolism to environmental changes in general, its potential role as a stress signal and mediator of environmental memory warrants more detailed studies.

### Using the knowledge on plant memory to predict plant responses to future climates

In parallel to experimental techniques, mathematical modelling is an extremely promising approach to guide research and answer such complex biological questions as unravelling the environmental memory of plants (Figure 2). Mathematical models are of great importance when the data are not complete or readily available, empowering our ability to predict or understand responses even in the absence of empirical results. It has been used successfully in the study of vernalization, where the complexity of the system made it highly desirable and where a good molecular understanding was available as a basis (see the ‘Molecular mechanisms involved in the regulation of plant memory’ section).

With the rising interest in transgenerational plasticity in recent years, there has also been a development towards considering plant memory in theoretical models of ecology and evolution. Theoretical developments integrating transgenerational memory and its given contribution to phenotypes into evolutionary models have taken a range of forms, from mechanistic models describing the inheritance of small RNAs (Silva *et al.* 2021) to general evolutionary models without mechanistic detail (Leimar and McNamara 2015; Dury and Wade 2020). For an extensive review of the general theory of non-genetic inheritance of traits, please see Bonduriansky *et al.* (2012).

It is not a new idea that non-genetic information can play a role in evolution (Waddington 1940, 1959), but it is only recently that formal models have explored more in depth when this is expected to be important and which role it plays under different circumstances. Theoretical models show that within- and transgenerational memory can work together to produce adaptive phenotypes, and transgenerational memory can speed up adaptation to new environments (Hoyle and Ezard 2012; Ezard *et al.* 2014). When the conditions and phenotypes of parents (often maternal individuals) are better predictors of future environments than conditions experienced by progeny themselves, models indicate that transgenerational memory (or long-term memory) is favoured (Dury and Wade 2020). Furthermore, models have shown that when transgenerational memory is not costly, it will evolve if environmental predictability is high enough (Figure 1), even when within-generation plasticity can help match the phenotype later in life (Kronholm 2022). When the probability of accurately predicting future environments is low, and the cost of memory is the same for both within- and transgenerational plasticity, within-generational plasticity is advantageous, and for some phenotypes, such as flowering control by vernalization or defence responses to pathogens, within-generational plasticity control would be preferable (Dury and Wade 2020). In addition, when several cues potentially act together, within- or transgenerational memory can evolve in concert and the phenotype can be expected to be determined by a weighted combination of the cues where the most accurate one is given the highest weight (Leimar and McNamara 2015). These model predictions of the adaptive advantages of different strategies in different conditions can shed light on the evolutionary trajectories that lead to them, and the underlying principle is illustrated in Figure 1. However, there is a greatly underutilized potential in developing this type of model into more predictive models for responses to future changes in the environment.

A different use of mathematical modelling is direct application in agriculture, rather than understanding. Models that can predict the plant’s response to its environment are instrumental for growers to plan and optimize their harvests. In line with this, some successful uses of mathematical modelling show that it is possible to make predictions of plant response and yield. Such models can be used to predict how crop yield might be affected by natural environment changes or by agricultural interventions, and they can even help identify adaptive traits. One large collection of models is APSIM (Holzworth *et al.* 2014), which has modules for various crop species and can simulate their biophysical processes in the context of agricultural systems. These models are already widely used and are very valuable for agriculture.

The above-mentioned models necessarily need to incorporate plant memory (so far within-generation memory) since it is not only the current environment that affects a plant’s response but also the history of the environment it has experienced. An example of this in model species is the control of flowering time through vernalization in the *Brassicaceae*, where there are several models that predict phenology (Wilczek *et al.* 2009) and gene expression for the key flowering gene *AtFLC* in *A. thaliana* or its homologs in other species (Aikawa *et al.* 2010; Satake *et al.* 2013; Antoniou-Kourounioti *et al.* 2018; Nishio *et al.* 2020). In these examples, the underlying molecular mechanisms of plants have been included in the predictive models to different degrees. Furthermore, these studies used field data to inform and test the models allowing them to make predictions beyond what was previously possible based on the understanding from only controlled conditions. This proved to be a powerful technique both for predictive power and for mechanistic model selection.

Mechanistic models have the advantage of giving valuable scientific insights that can be useful in other systems (e.g. Angel *et al.* 2011; Berry *et al.* 2017; Holoch *et al.* 2021). They can also be used to identify breeding targets for the development of weather-proof crops. On the other hand, mechanistic models need to make assumptions and rely on existing knowledge of the molecular processes controlling the plant response to multiple environmental factors (e.g. temperature, light, rainfall, soil nutrients, etc.). In many cases and for many crop species, this level of knowledge is not yet available, and a considerable amount of work is still needed to reach that level.

To make predictions in cases with limited mechanistic understanding, the opposite approach has also been taken, which is to use no mechanistic knowledge/assumptions but let the data reveal the relationships between environmental conditions and plant response—the machine learning approach (van Klompenburg *et al.* 2020). In this case, even though good predictive power can be achieved from a large volume of data, it is not possible to learn about the underlying mechanism. For example, a recent study correlated the flowering time of bulbous perennials with various environmental conditions and found that snow depth anomaly is best correlated (Jánosi *et al.* 2020). Though this is unlikely to be a direct relationship, it could suggest that snow depth anomaly is a good proxy for the most appropriate combination of other environmental parameters that directly affect the plant. Another study combined the two approaches (mechanistic modelling and artificial neural networks) to predict an idealized expression of flowering time genes (L. Zhao *et al.* 2020a). They found that combined long-term temperature and short-term light (day lengths) data gave the best predictive power, highlighting the importance of a memory mechanism for within-generation temperature information.

Machine learning is very powerful for making predictions based on past data and trends, but it could become less reliable in future climates if weather patterns are altered with climate change. Furthermore, both machine learning and the more traditional approaches need a lot of data for development and parameterization, and in many regions, or for specific crops or species, such data are not available, the available data are in the wrong format, or they are of insufficiently good quality (Filippi *et al.* 2019; Kephe *et al.* 2021). A way to improve these models is to supplement incomplete datasets by combining field-based data with controlled experiments (Kephe *et al.* 2021). Another way to potentially improve machine learning models is by incorporating biological understanding into these, and this is starting to become more popular with some promising results, e.g. in the case of a sorghum yield prediction model (Fozard 2021).

Models become extremely useful when their predictive ability is enhanced by empirical data that more precisely point to outcomes under a certain set of future environmental conditions. Data derived from memory experiments can help achieve this goal. For example, adding environmental effects to genomic-selection models (G × E) have been attempted to improve the successful breeding of complex traits (Crossa *et al.* 2022). However, incorporating information on effects of environments across generations (G × E_G1_ × … × E_G*n*_) in mechanistic models poses a real challenge from the computational point of view, although they may inform on how those complex traits may render adaptive responses in the face of different environmental scenarios (Lawson *et al.* 2015). Furthermore, while it is potentially feasible to add more parameters to build more precise models, not only to predict plant responses in agricultural ecosystems but also in those to help with conservation programmes, these efforts should be accompanied by building accessible processing capacity and supporting the acquisition of sufficient experimental data to constrain those parameters. In sum, we believe this is an incredible opportunity to build multidisciplinary collaborative research efforts.

Box 1. **The Way Forward**

The growing literature on plant memory, and the urgency for understanding biological processes in a context of climate change, emphasize the need to further our knowledge on long-term plant responses to environmental changes. There are some evident research paths to take and to answer unresolved questions on this topic; however, we would like to stress some points and provide some suggestions for approaching the exploration of plant memory to have a higher impact. No matter the directions taken in research, it is clear that establishing multidisciplinary efforts can be the best way forward to disentangle the plant memory secrets.

*Considerations for eco-evo research*:

- Much of the research mentioned in this paper was performed under controlled growth conditions with single-cue changes. Although controlled conditions allow us to understand individual contributions of environmental cues, analysing plant memory responses in more complex and realistic conditions may improve our interpretation with greater implications for the ecological and evolutionary processes in future climatic scenarios.
- As responses to the environment may be strongly influenced by their interaction with the genetic or epigenetic environment within and across generations, it is advisable to incorporate different (epi)genotypes and accessions/populations to assess the relevance of (epi)G × E interactions on plant memory.
- The importance of plant memory for short-lived species might be considered obvious, especially in a short timescale. Notwithstanding, our understanding of plant memory for long-lived species is profoundly lacking. As environmental memory can also be beneficial for long-lived species, funding efforts to perform long-term studies is extremely necessary.

*Considerations for the study of the genetic, epigenetic and metabolic mechanisms underlying plant memory*:

There are many questions about the mechanisms involved in establishing, transmitting and dissipating plant memory. The nature of such mechanisms and the interactions among them imply a level of complexity that is difficult to summarize in a text box. However, we believe there are some outstanding challenges from the molecular biology point of view:

- It is interesting to inquire how the different kinds of molecular modifications feed into a system that allows plants to have increased plasticity, especially when this plasticity persists across developmental transitions and across generations. For example, how long do epigenetic changes mediating plant memory persist? What is the contribution of genetic variation to the perpetuation of lasting molecular functions? Is dissipation of plant memory a reset of molecular mechanisms to pre-environmental signal levels?
- As the metabolism is an extremely powerful means for plants to adjust and adapt to changing environments, the contribution of this molecular component to plant memory could help us disentangle other mechanisms involved.

*Considerations for the modelling of plant memory:*

- Putting together empirical data from natural and controlled conditions can provide mathematical models with more powerful and realistic insights on the prediction of plant responses to future climatic scenarios.
- Developing more efficient and precise models that incorporate information across multiple generations might benefit from access to computational processing power and appropriate data to parameterize these models. This will require the joint work of computer scientists and biologists, thus establishing multidisciplinary efforts may be the only way to produce substantial advancements.

## Concluding Remarks

The complexity of plant memory is extremely intriguing, although expected. As a result of its importance in affecting eco-evo trajectories, plant memory must be tightly regulated through multiple fail-safe, plastic, and sometimes redundant mechanisms. Investigating how plant memory is established, transmitted, maintained and dissipated requires a multilevel effort that may be constrained by researchers’ access to cutting-edge techniques. However, multidisciplinary approaches may facilitate our understanding of the mechanisms, their consequences at eco-evo levels, and their realized predictive and biotechnological potential. The problem becomes considerably more important and complex when additional organisms in the rhizo- and phyllosphere are involved, as it is known that the latter may contribute and direct plant memory responses (Ueno *et al.* 2021). Altogether, plant environmental memory research is an unprecedented opportunity to learn about the natural world.

## Data Availability

No new data were generated for this review article.

## Notes

**Phenome, Genome and Environment. Chief Editor:** Colleen Doherty
